# Predictors of gait speed and its change over three years in community-dwelling older people

**DOI:** 10.18632/aging.101365

**Published:** 2018-01-20

**Authors:** Daniela Pinter, Stuart J. Ritchie, Thomas Gattringer, Mark E. Bastin, Maria del C. Valdés Hernández, Janie Corley, Susana Muñoz Maniega, Alison Pattie, David A. Dickie, Alan J. Gow, John M. Starr, Ian J. Deary, Christian Enzinger, Franz Fazekas, Joanna Wardlaw

**Affiliations:** 1 Department of Neurology, Medical University of Graz, Graz, 8036, Austria; 2 Centre for Cognitive Ageing and Cognitive Epidemiology, University of Edinburgh, Edinburgh, EH8 9YL, UK; 3 Department of Psychology, University of Edinburgh, Edinburgh, EH8 9YL, UK; 4 Brain Research Imaging Centre, University of Edinburgh, Edinburgh, EH4 2XU, UK; 5 Centre for Clinical Brain Sciences, University of Edinburgh, Edinburgh, EH4 2XU, UK; 6 Department of Psychology, Heriot-Watt University, Edinburgh, EH14 4AS, UK; 7 Alzheimer Scotland Dementia Research Centre, University of Edinburgh, Edinburgh, EH8 9YL, UK; 8 Division of Neuroradiology, Vascular and Interventional Neuroradiology, Department of Radiology, Medical University of Graz, Graz, 8036, Austria; 9 UK Dementia Research Institute at the University of Edinburgh, Edinburgh, EH8 9YL, UK

**Keywords:** gait, white matter hyperintensity volume, brain volume, grip strength, body mass index, aging

## Abstract

We aimed to assess whether and how changes in brain volume and increases in white matter hyperintensity (WMH) volume over three years predict gait speed and its change independently of demographics, vascular risk factors and physical status. We analyzed 443 individuals from the Lothian Birth Cohort 1936, at mean age 73 and 76 years. Gait speed at age 76 was predicted by age, grip strength and body mass index at mean age 73, three-year brain volume decrease and WMH volume increase, explaining 26.1% of variance. Decline in gait speed to age 76 was predicted by the same five variables explaining 40.9% of variance. In both analyses, grip strength and body mass index explained the most variance. A clinically significant decline in gait speed (≥ 0.1 m/s per year) occurred in 24.4%. These individuals had more structural brain changes. Brain volume and WMH changes were independent predictors of gait dysfunction and its three-year change, but the impact of malleable physical factors such as grip strength or body mass index was greater.

## Introduction

Aging is associated with an overall decline in lung Gait and balance disturbances are major concerns for older people and have been related to multiple factors and disorders [1–5]. Cerebral small vessel disease (SVD) has been particularly implicated in the etiology of gait problems [6–8]. In a cross-sectional study of community-dwelling healthy older people we found that brain volume and white matter hyperintensity (WMH) volume were each independently associated with gait speed [9]. Furthermore, WMH burden was the major contributor for gait impairment within a global SVD score, comprising WMH, microbleeds, lacunes and enlarged perivascular spaces [9]. Associations between the same brain variables and chair-stands or balance function were limited, possibly due to either a low impact of SVD or the low sensitivity of these applied tests in healthy older people.

Studies investigating the role of SVD in a longitudinal manner showed that initial WMH burden was associated with the subsequent development and severity of gait and balance dysfunction [7,10–16].

Only a few studies have investigated the association of WMH progression with increasing gait or mobility dysfunction [10,17,18]. In one study of 104 cognitively intact individuals aged 65 and older, WMH progression was associated with decreased gait performance over time, but no association with balance function was reported [17]. By contrast, in a study of 77 community-dwelling individuals aged 75 and older, WMH progression correlated with decreased mobility performance changes assessed by the chair-stands test, but not with gait [18]. A large longitudinal population-based study in 225 individuals aged 60 to 86 showed that WMH increase and brain atrophy were associated with gait decline [10]. These three previous longitudinal studies included individuals with a wide age-range of several decades and highlight how multiple variables, such as age, infarcts or brain volume, may modify associations between WMH increase and gait decline [10,17].

Therefore, we attempted to complement this information in a comprehensive analysis of longitudinal data from the Lothian Birth Cohort 1936 (LBC1936) which comprises generally healthy community-dwelling older people with a narrow age range. Based on our cross-sectional analysis [9], we focused specifically on the association between gait speed and changes in brain volume and in WMH volume, in addition to demographics, vascular risk factors and physical status.

## RESULTS

### Sample characteristics

Table 1 gives information on the demographics, physical, vascular risk factor variables, gait and balance function and MRI characteristics of the study sample at baseline and follow-up. Height and grip strength decreased over the three years of follow-up. The number of individuals reporting leg cramp, arthritis, high blood pressure, diabetes and high cholesterol increased from baseline to follow-up.

**Table 1:** Demographics, physical variables, vascular risk factors, gait and balance function and MRI characteristics of the study sample.

**Demographics**	**Baseline N=443**	**Follow-Up**	
Sex, male, N (%)	244 (55.10)		
Age in years	72.52 (0.69)	76.29 (0.65)	<0.001
**Physical factors**	**Baseline N=443**	**Follow-Up**	**p**
Height (FUP N= 442)	166.82 (8.98)	166.22 (8.92)	<0.001
Grip Strength (FUP N= 442)	29.01 (9.23)	27.69 (9.53)	<0.001
Leg cramp, N (%)	169 (38.10)	204 (46.00)	<0.001
Arthritis, N (%)	206 (46.50)	215 (48.50)	<0.001
**Vascular risk factors**	**Baseline N=443**	**Follow-Up**	**p**
Smoking (current), N (%)(FUP N= 442)	30 (6.77)	28 (6.33)	0.480
High Blood Pressure, N (%)(FUP N= 442)	219 (49.40)	249 (56.33)	<0.001
Diabetes, N (%)	42 (9.50)	55 (12.40)	<0.001
High Cholesterol, N (%)(FUP N= 440)	184 (41.50)	211 (47.95)	<0.001
BMI (FUP N= 442)	27.67 (4.07)	27.62 (4.26)	0.486
**Gait Speed and Balance function**	**Baseline**	**Follow-Up**	**p**
6 meter walk time (sec)	4.15 (1.01)	4.56 (1.27)	<0.001
Gait speed (m/sec)	1.52 (0.34)	1.40 (0.35)	<0.001
Chair-Stands (sec) (FUP N=420)	13.11 (3.77)	13.01 (4.13)	0.607
Standing balance score= 4; N (%) (FUP N=441)	391 (88. 30)	367 (83.22)	<0.001
Gait impairment: speed < 1.0 m/s	N=22 (5.0%)	N=43 (10.4%)	
**MRI characteristics**	**Baseline**	**Follow-Up**	
Brain Volume (cm^3^)	995.46 (92.50)	976.53 (90.53)	<0.001
WMH Volume (cm^3^)	11.75 (11.73)	15.62 (14.52)	<0.001
Old infarcts	53 (12.0%)		
Lacunes (1 or more)	25 (5.6%)		
PVS (grade 2-4 in BG)	176 (39.7%)		
CMB	49 (11.1%)		

Sample characteristics are presented as mean and standard deviation (SD in brackets), or number of individuals (N) and percentage (%). FUP = 3 year follow-up, N = number of participants.

BG= basal ganglia, CMB= cerebral microbleeds, PVS= perivascular spaces

Mean gait speed decreased significantly across the three years and the magnitude of change was on average about one-third of a standard deviation. At baseline (mean age 73), gait impairment with a walking speed below 1.0 m/s was observed in 5.0% (N=22) and at follow-up (mean age 76) in 10.4% (N=43). Gait speed declined clinically significantly (≥ 0.1 m/s per year) in 108 participants (24.4%). Performance on the chair-stands test did not significantly change over the three years of follow-up, but performance on the standing balance test significantly decreased (*p* < 0.001).

Brain volume significantly decreased and WMH volume significantly increased across the three years (Table 1). Brain volume change and WMH progression did not correlate (*r* = -0.032; *p* = 0.502).

### Prediction of gait speed at age 76

The regression model revealed that age, grip strength and BMI at baseline, brain volume decrease and WMH increase independently contributed to the prediction of gait speed at age 76, explaining in total 26.1% of variance. Age, grip strength and BMI at baseline were the strongest predictors, explaining together 23% of the variance in gait speed, whereas volume changes of the brain and WMH added little (1.6 and 1.5% respectively) to explaining the variance in gait speed. For further details please see Table 2.

**Table 2:** Results of hierarchical regression models to predict gait speed at mean age 76.

**Gait speed (m/sec)**		**R^2^**	**β (95% CI)**	***βj***	**p**
Age*		10.6	-0.082 (-0.123 to -0.041)	-0.163	<0.001
Grip Strength*		17.3 (6.7)	0.013 (0.007 to 0.018)	0.339	<0.001
BMI*		23.0 (5.7)	-0.019 (-0.026 to -0.012)	-0.223	<0.001
BV Change		24.6 (1.6)	3.4000 (1.168 to 5.633)	0.125	0.001
WMH Change		26.1 (1.5)	-16.618 (-26.931 to -6.306)	-0.132	0.002

Adjusted R^2^ (explanation of variance), betas (β), and confidence intervals (CI) and standardized beta-values (*βj*) are presented for significant findings only. Incremental explanation of variance is shown in brackets as delta (∆) of adjusted R^2^. BV = brain volume, WMH = white matter hyperintensity volume. N=443

*at baseline with age 73.

### Prediction of changes in gait speed

The regression model revealed that gait speed, grip strength and BMI at baseline, brain volume decrease and WMH increase independently contributed to changes in gait speed between age 73 and 76, explaining in total 40.9% of variance. Gait speed at baseline was the strongest predictor (33.1%) and volume changes of the brain and of WMH added little (1.3% and 1.1% respectively) to explaining the variance in gait speed (Table 3). Including duration of follow-up in the regression analyses did not change the results (data not shown).

**Table 3:** Results of hierarchical regression models to predict changes in gait speed.

**Gait speed (sec)**	**R^2^**	**β (95% CI)**	***βj***	**p**
Gait speed (sec)*	33.1	0.449 (0.364 to 0.533)	0.434	<0.001
Grip Strength (kg)*	36.7 (3.6)	0.008 (0.003 to 0.013)	0.221	<0.001
BMI*	38.5 (1.8)	-0.013 (-0.019 to -0.006)	-0.149	<0.001
BV Change	39.8 (1.3)	3.101 (1.104 to 5.099)	0.114	0.002
WMH Change	40.9 (1.1)	-14.029 (-23.265 to -4.794)	-0.111	0.003

Adjusted R^2^ (explanation of variance), betas (β), and confidence intervals (CI) and standardized beta-values (*βj*) are presented for significant findings only. Incremental explanation of variance is shown in brackets as delta of adjusted R^2^. BV = brain volume, WMH = white matter hyperintensity volume. N=443

* at baseline with age 73.

### Differences of individuals with vs. without clinically significant gait decline

Individuals with a clinically significant gait decline between 73 and 76 years as defined by a decrease of gait speed of >-0.1 m/s per year had walked faster and were slightly younger at baseline but showed no other significant differences in any of the variables assessed (Table 4). The time interval between their baseline and follow-up assessment was slightly longer than that of individuals whose change in gait speed did not reach the level of a significant decline. Individuals with clinically significant changes in gait speed showed bigger changes in gait speed and also showed larger changes at the chair-stands test. Furthermore, individuals with a clinically significant gait decline showed a greater reduction of normalized brain volume and increase in WMH volume. Changes in all other variables were non-significant (Table 4). Comparable results were obtained when performing these analyses after exclusion of individuals with gait impairment at baseline (N=22).

**Table 4:** Differences at baseline and differences of change of participants with vs without a priori defined significant gait decline (>0.1 m/s per annum).

**Baseline age 73**			**p**	**Changes**		**p**
	**Decline** **N =108 (24.4%)**	**No Decline** **N =335 (75.6%)**		**Decline**	**No Decline**	
Sex/male, N (%)	68 (62.96)	176 (52.54)	0.060	NA	NA	
Age	72.23 (0.90)	72.63 (1.07)	**0.004**	3.82 (0.11)	3.79 (0.16)	**0.015**
**Physical factors**						
Height	168.40 (12.75)	166.49 (13.60)	0.131	-0.50 (1.28)	-0.50 (1.25)	0.552
Grip Strength	30.50 (15.88)	29.00 (15.50)	0.394	-1.75 (5.50)	-1.00 (4.38)	0.293
Leg cramp, N(%)	39 (36.11)	130 (38.81)	0.650	21 (19.44)	59 (17.61)	0.448
Arthritis, N(%)	51 (47.22)	155 (46.27)	0.912	8 (7.41)	32 (9.55)	0.219
**Risk factors**						
Smoking, N(%)	9 (8.33)	21 (6.29)	0.615			
HBP, N(%)	49 (45.37)	170 (50.75)	0.376	7 (6.48)	32 (9.55)	0.602
Diabetes, N(%)	12 (11.11)	30 (8.96)	0.571	5 (4.63)	9 (2.69)	0.517
HChol, N(%)	45 (41.67)	139 (41.50)	0.975	15 (13.89)	33 (9.85)	0.481
BMI	27.53 (4.96)	27.19 (4.84)	0.449	0.09 (1.39)	0.02 (1.72)	0.387
**Gait and Balance**						
6 meter walk time (sec)	3.60 (0.83)	4.33 (0.99)	**<0.001**	1.59 (0.87)	0.23 (0.69)	**<0.001**
Gait speed (m/sec)	1.72 (0.52)	1.45 (0.33)	**<0.001**	-0.46 (0.22)	-0.03 (0.27)	**<0.001**
Chair-stands(sec)	12.07 (5.02)	13.04 (4.62)	0.051	0.29 (4.52)	-0.27 (4.29)	**0.002**
Standing score=4, N(%)	96 (88.9)	295 (88.1)	0.725	5.56% decrease	4.81% decrease (N=333)	0.685
**MRI-markers**						
Brain Volume*	68.94 (2.79)	69.24 (2.76)	0.173	-1.46 (0.02)	-1.07 (0.01)	**0.003**
WMH Volume*	0.55 (0.86)	0.51 (0.73)	0.454	0.26 (0.01)	0.17 (0.01)	**0.024**

Sample characteristics are presented as median and interquartile range (IQR in brackets) if not otherwise specified. Changes for nominal variables are indicated as number of additional participants with a present physical or risk factor at follow-up.

HBP = high blood pressure, HChol = high cholesterol, *Normalized by intracranial volume in %

### Sensitivity analysis excluding individuals with a history of stroke

Out of 443 individuals, 28 reported a history of stroke at baseline during the medical interview. In the entire sample, history of stroke did not contribute to the prediction of decline in gait impairment (*p* = 0.106). Sensitivity analysis for 415 individuals without a self-reported history of stroke revealed identical findings showing that age (*p* = 0.021; *R^2^* = 5.0%); grip strength (*p* < 0.001; *∆R ^2^*= 9.7%), BMI (*p* < 0.001; *∆R^2^* = 5.0%), brain volume change (*p* = 0.006; *∆R^2^* = 1.4%) and WMH increase (*p* = 0.001; *∆R^2^* = 1.9%) predicted gait speed at mean age 76, explaining 23.0% of variance. Results for changes in gait speed were identical for the sample excluding those with a history of stroke (*R^2^* = 47.1%), (gait speed at baseline *p* < 0.001; *R^2^* = 42.3%, grip strength *p* < 0.001; *R^2^* = 2.2%, BMI *p* = 0.005; *R^2^* = 0.5%, brain volume change *p*= 0.003; *R^2^* = 1.1%, WMH volume change *p* = 0.004; *R^2^* = 1.0%).

## DISCUSSION

This three-year longitudinal study shows that even in generally healthy community-dwelling individuals with a narrow age-range, changes in brain and WMH volumes over three years were significant and independent predictors of gait speed and changes in gait speed. However, the comparatively larger impact of malleable physical factors such as grip strength or BMI is noteworthy and raises the possibility of reducing gait dysfunction in older adults with targeted interventions and of predicting gait dysfunction with simple office tests of grip strength.

Our study more than doubles the data from previous longitudinal investigations on the influence of WMH progression on gait and balance function [10,17] and extends the knowledge base by including baseline gait performance, multiple vascular risk factors and physical variables. The findings suggest that in community-dwelling older adults physical variables such as grip strength and BMI might be more important than brain structural changes, but that both contribute to change in gait speed. We observed that higher grip strength was associated with faster walking at mean age 76 and explained 6.7% of gait impairment and 3.6% of changes in gait speed. Grip strength has been used as a proxy for global physical function, sarcopenia and frailty [19,20]. The promotion of muscle strength in older people might therefore be a target to reduce gait impairment, although further studies are needed in this context. Previous cross-sectional findings note that physical activity might have the potential to reduce the risk of mobility impairment in the older people with mild to severe WMH burden [21] and a recent meta-analysis including 42 studies reports that exercise interventions can increase gait speed and help to slow the decrease of gait speed or delay its onset [22].

Furthermore, a higher BMI was related to slower walking at mean age 76. This finding is in line with findings that increasing BMI over 25 years is associated with worse late-life gait speed [23]. A recent study showed that a high BMI scores (over 30) is associated with a higher risk of developing mobility impairment [24]. Two longitudinal studies assessing the relationship between WMH increase and gait impairment included BMI in their regression models, but did not report if and to what extent BMI predicted gait impairment [10,18]. In addition, physical fitness, as measured by grip strength, lung function and six meter walk time, has been shown to be a protective factor for structural brain ageing in the LBC1936 [25].

This study also extends several previous longitudinal studies which investigated the impact of WMH progression on gait impairment and decline by minimizing the confounding effects of age [7,10,11,14,17].

Callisaya et al. [10] highlight that the relative contribution of atrophy and WMH progression strengthens the causal relationship between structural brain changes and gait decline. However, atrophy [26], the burden and increase of WMH [27], the extent and decline of gait impairment [3,4] and their associations are all influenced by increased age [10]. Hence, a major strength of our study is the investigation of a large sample with a narrow age range.

Both degenerative and vascular processes have been associated with age-related gait impairment in cross-sectional studies [28]. Interestingly and in line with the only previous longitudinal study assessing brain and WMH volume change in relation to gait decline [10], we found similar but independent contributions of a decrease in brain volume and of WMH progression on gait impairment and decline. In this context it is also noteworthy that the decrease in brain volume and progression of WMH in our cohort were comparable to prior studies [10,18,29] of individuals with this age-range.

It is not unexpected that gait speed at baseline is associated with gait function three years later. In fact baseline gait speed was by far the strongest, positive predictor of gait speed at follow up. However, when comparing individuals with and without a clinically significant gait decline as defined by an annual reduction of gait speed of > 0.1 m/s [30,31], i.e. > 0.3 m/s in our study, those with a significant decline had walked faster at baseline. While this may appear implausible at first, it probably attests to the fact that decline in gait speed is a not a fully linear phenomenon. Furthermore, individuals with high gait speed at baseline have the largest possibility for change, therefore the regression to the mean phenomenon should also be considered in this context [32]. Other significant differences between individuals without and with a significant decline in gait speed were a larger decrease in brain volume and increase in WMH volume in the latter. Individuals with a significant decline in gait speed also had a significantly but only slightly longer time interval to follow up assessment. However, including the follow-up duration in the regression analysis did not influence the results of the regression analysis.

Since both stroke [33] and the occurrence of WMH have been associated with gait impairment in older people, we performed sensitivity analysis excluding individuals reporting a history of stroke. In line with a previous study, history of stroke did not affect the impact of WMH change on gait speed and decline [11].

Given the major impact of gait impairment for older adults regarding increased mortality, risk of developing dementia or cardiovascular disease, risk of falling and functional independence predicting early gait dysfunction and decline is highly desirable [3,30]. In this regard our findings suggest that for prediction of gait function and decline in healthy older people a simple assessment of physical function and of vascular risk factors might be sufficient. Although MRI-markers such as changes in brain volume and WMH volume independently add to the explanation, the expense of an MRI-measurement might not be essential regarding prediction of gait in large samples.

When interpreting our findings the specific setting of a healthy community-dwelling cohort with a follow-up duration of only 3 years and thus only small changes in all variables needs to be considered. The fact that we were able to observe significant associations of brain morphologic and physical factors with gait speed and its decline therefore attests to the robustness of this relation. At the same time this setting entails some limitation as we may have not been able to detect more subtle relationships with other variables which could impact gait speed. Likewise more sensitive and robust tests would be needed for similar analysis in regard to balance function.

## METHODS

### Participants

Data from community-dwelling older people who were members of the LBC1936 were analyzed [34,35]. More detailed information of the recruitment and study procedures can be found in Deary et al. [34,35]. We included only those individuals who underwent comprehensive risk factor assessment and gait and balance assessment and brain MRI acquisition at age 73 (age range = 70.96 to 74.16 years; Wave 2 of the LBC1936 study) and 76 (age range = 74.73 to 77.75 years; Wave 3). Eight participants were excluded from further analyses due to a diagnosis of Parkinson’s disease (N=1, Wave 2; N=2 Wave 3) and/or dementia (N=6, Wave 3), rendering a final sample of 443 individuals.

### Standard protocol approvals, registrations, and patient consents

Ethics permission for the study protocol was obtained from the Multi-Centre Research Ethics Committee for Scotland (MREC/01/0/56) and from Lothian Research Ethics Committee (LREC/2003/2/29). The research was carried out in compliance with the Helsinki Declaration. All assessments were performed in accordance with relevant guidelines and regulations. All participants gave written, informed consent.

### Vascular risk factor assessment and physical examination

An extensive description of all variables obtained for the LBC1936 study can be found in Deary et al. [35] for clinical and Wardlaw et al. for imaging variables [36]. All participants underwent a medical interview and physical examination (e.g. height and weight to calculate Body Mass Index (BMI kg/m^2^)). Disease history (e.g. self-reported history of stroke), physical variables (e.g. self-reported leg cramp when walking or in bed at night, self-reported arthritis) and vascular risk factors (e.g. smoking, self-reported high blood pressure, self-reported diagnosis of diabetes, self-reported high cholesterol) were obtained in a structured interview. As part of the physical examination we also obtained grip strength in the right and left hand using a North Coast Hydraulic Hand Dynamometer (JAMAR) and used overall grip strength in kg (best of three trials of the right and left hand) for our analyses.

### Assessment of gait and balance function

Gait speed was assessed by the six-meter walk test, a common assessment used in research studies to access physical function [37]. Participants were asked to walk as quickly as possible with the use of a cane or walker if appropriate. Gait speed was measured in meters/second. Gait impairment was defined as a gait speed < 1.0 m/s, as this cut-off was suggested to identify persons with a high risk of health-related lower extremity limitations and hospitalization [30,38] and clinically significant gait decline was defined as >-0.1 m/s per annum, as this decline was related to survival rates in a large pooled analysis of nine cohort studies [30,31]. Furthermore, two subtests (chair-stands and standing balance) of the Short Physical Performance Battery were applied as described earlier [39]. In brief, the chair-stands test assesses how long (seconds) it takes the participant to stand up and sit down as quickly as possible five times without stopping. The test of standing balance includes side-by-side, semi-tandem, and tandem stands which are scored from 1 to 4 points, reaching 4 points indicates the best standing balance performance.

### Brain MRI acquisition

The brain imaging protocol for the study has been described previously [36]. All participants were scanned on a General Electric 1.5 T clinical MRI scanner (Signa Horizon HDx) operating in research mode. For this study, we used axial T2-, T2*-, fluid-attenuated inversion recovery (FLAIR) and T1-weighted sequences.

### Assessment of brain volume and WMH volume

Intracranial (ICV), whole brain, and WMH volumes were assessed in mL using a validated multispectral image processing method that combines T1-, T2-, T2*-, and FLAIR-weighted MRI sequences for segmentation [40]. All sequences were coregistered and tissue volumes estimated by cluster analysis of voxel intensities. WMH masks were manually edited by following Standards for Reporting Vascular changes on neuroimaging guidelines and using 3D-mask editing software, Multi-Image Analysis GUI (MANGO; http://ric.uthscsa.edu/mango/). Editing was overseen by a neuroradiologist (JMW). We manually checked all segmented images for accuracy blinded to all clinical details, corrected errors, and excluded imaging-detected cortical and subcortical infarcts from WMH manually. Brain tissue volume and WMH volume normalized by ICV were used for the analyses.

### Statistical analysis

We used the Statistical Package of Social Science (IBM SPSS Statistics 23, SPSS Inc., Chicago, Illinois, USA) for paired t-tests, nonparametric analysis (e.g. McNemar and Wilcoxon Rank), correlation and regression analyses.

A hierarchical linear regression model was used to assess whether gait speed at mean age 76 was independently predicted by changes in normalized brain volume and an increase in normalized WMH volume in addition to demographics (sex, age), baseline physical factors (height, grip strength, leg cramp, arthritis), and baseline vascular risk factors (smoking, diabetes, high blood pressure, high cholesterol, BMI). To assess whether brain volume and WMH increase independently predicted changes in gait speed, the same model was applied including gait speed at baseline (mean age 73) in the first step.

We checked for fulfilment of different assumptions for the regression analyses (e.g. linearity, homoscedasticity, auto-correlation (Durban-Watson-test), multicollinearity (Tolerance and Variance Inflation Factor)). Betas and confidence interval (CI), standardized beta-values (βj), adjusted R^2^ (explanation of variance) and delta (Δ) adjusted R^2^ (displaying incremental explanation of variance) in percent are presented for each model in the results section. The adjusted R^2^ reflects R^2^ that has been adjusted for the number of predictors in the model. R^2^ tends to optimistically estimate the fit of the linear regression. It always increases as the number of predictors increase in the model. Therefore adjusted R^2^ attempts to correct for this overestimation. Standardized beta coefficients put all of the variables on the same scale, and allows a comparison of the magnitude of the coefficients to see which one has the larger effect size. The level of significance was set at 0.05.
